# Association between hearing loss, depression, anxiety, and social isolation in middle-aged and older outpatients in Afghanistan

**DOI:** 10.5339/qmj.2024.57

**Published:** 2024-10-15

**Authors:** Mohammad Hussain Hamrah, Ahmad Tareq Hamrah, Mohammad Hassan Hamrah, Leusa Ozturk, Elaha Somaya Ghafary, Toba Dahi, Mohammad Hashem Hamrah

**Affiliations:** 1 Department of Gastroenterological Surgery, Nagoya University Graduate School of Medicine, Nagoya, Japan; 2 Curative Clinic, Andkhoy, Afghanistan *Email: hashem.sahab@gmail.com; 3 School of Medicine, Tehran University of Medical Sciences, Tehran, Iran; 4 Department of Public Health and Health Systems, Nagoya University Graduate School of Medicine, Nagoya, Japan; 5 Faculty of Medicine, Bursa Uludag University, Bursa, Turkey; 6 Department of Periodontics, Kabul University of Medical Sciences, Kabul, Afghanistan; 7 Faculty of Dentistry, AbantIzzet Baysal University, Bolu, Turkey

**Keywords:** Hearing loss, social isolation, depression, anxiety, Afghanistan

## Abstract

**Background:**

Hearing loss (HL) is a major public health problem that is significant for mental health and physical conditions. HL may be a potentially modifiable risk factor for poorer mental health outcomes. The study aimed to explore the association between HL, social isolation, anxiety, and depression.

**Methods:**

A cross-sectional study was conducted on a sample of consecutive patients aged 50 years or older (*n* = 226) from February 2023 to August 2023. We used regression models to assess the association between HL and the odds of social isolation, anxiety, and depression.

**Results:**

There are more female participants with HL (58.7% vs. 41.3%) than male individuals with HL. HL was found to have a statistically significant association with smoking (*p* = 0.001), and HL showed significant associations with anxiety and depression (*p* = 0.008 and *p* = 0.011, respectively). A significant association was also observed between HL and social isolation (*p* = 0.016). HL was associated with diabetes mellitus (*p* = 0.006) and hypertension (*p* = 0.008). Participants with HL were more likely to have depression (OR = 2.16; 95% CI: 1.05–4.44), social isolation (OR = 2.87; 95% CI: 1.44–5.70), and anxiety (OR = 2.62; 95% CI: 1.21–5.67) compared to participants in the no hearing loss (No-HL) group.

**Conclusions:**

This study shows that HL is consistently associated with poorer mental health symptoms and poor social isolation. Although additional evidence is necessary, it is plausible that addressing HL would alter this trajectory.

## 1. Introduction

In 2019, approximately 1.57 billion people in the world had hearing loss (HL), and this number is expected to increase to 2.45 billion by 2050.^1^ The World Health Organization estimates that untreated HL imposes an annual cost of US$980 billion on the global economy. This includes health sector expenses (excluding the cost of hearing devices), expenditures on educational support, productivity losses, and societal costs.^2^ The prevalence of HL increased with age; among older adults (older than 60 years), more than one in four people are affected by HL.^3^ It is estimated that approximately 1.3 billion people worldwide suffer from age-related or adult-onset HL.^4^ Low- and middle-income countries have a high prevalence of HL.^5^ HL is a major public health problem that can have severe physical, social, cognitive, economic, and emotional impacts on people’s quality of life.^6^

Depression is a prevalent mental health issue in the elderly population.^7^ The global prevalence of depressive disorder among older adults has been estimated to range between 10% and 20%^8^, while the prevalence of anxiety is between 14.2% and 39.4%.^9^ Depression and anxiety are frequent comorbidities.^7^ HL was associated with neuropathological *changes* and had a profound impact on auditory perception and mood.^10^ The most important risk factors for HL are male gender, genetic predisposition, congenital deafness, aging, infectious diseases (e.g., meningitis, chronic ear infections), ototoxic drugs, exposure to excessive noise, and increasing age.^11^

HL tends to disrupt interpersonal communication, and it can lead to social isolation.^12^ HL is associated with increased depression and social isolation in older adults^13^, and the relationship between HL and depression decreased after adjustment for social engagement.^14^ Many patients seen by hearing healthcare providers experience some form of anxiety, which can be exacerbated by the presence of HL or the prospect of treatment for that loss.^15^ There is a lack of specific information on the prevalence of depression, anxiety, and social isolation among older adults in Afghanistan. Comprehensive provincial statistics are currently unavailable due to prolonged conflict and instability.^16^ Our previous study showed a high prevalence of depression and anxiety in our outpatient clinic in Afghanistan.^17^ We hypothesized that individuals with HL would experience higher levels of social isolation, anxiety, and depressive symptoms compared to those without HL. The aim of this study was to determine the association between HL, social isolation, anxiety, and depression among patients visiting an outpatient clinic in Andkhoy, Afghanistan. The results of this study may be valuable in increasing awareness and understanding among health professionals and clinical practitioners regarding depressive symptoms, anxiety, and social isolation associated with hearing status.

## 2. Methods

### 2.1. Participants and data collection

From February 2023 to August 2023, we conducted a cross-sectional study at the Curative Clinic in Andkhoy, Afghanistan, an outpatient clinic. The study included 226 consecutive patients who were at least 50 years of age and older. The Curative Clinic is a referral center located in Andkhoy, in the northern region of Afghanistan.^18^ Informed consent was obtained from each study participant prior to their participation in the research. This study was approved by Faryab Regional Hospital’s Scientific Review Committee with a code of 1122-709.

During clinic visits, trained doctors conducted face-to-face interviews as part of the study protocol to assess patients. The data collection involved using a two-part questionnaire. The first part gathered information on demographic and socioeconomic characteristics, including age, sex, educational level (illiterate, primary, or private education, secondary, high school, or higher education), marital status (single, married, or other), occupation (employed, unemployed, housewife, or farmer), and details related to physical activity, diet, obesity, management of hypertension and diabetes, and smoking. The study variables are detailed elsewhere.^18^ Information on hearing impairment was obtained through self-reports during the initial assessment. Participants were categorized into two groups based on their responses (yes or no) to the question, “Do you have a hearing impairment?” Participants were further inquired about the state of their hearing, with response options ranging from “excellent” to “good,” “poor,” and “unable to hear.” Those indicating “excellent” or “good” were classified as having good hearing, while those selecting “poor” or “unable to hear” were categorized as having HL. A HL of up to 20 decibels below the hearing threshold is still within the range of normal hearing. HL can be categorized by severity as follows: (a) mild HL: 20–40 decibels, (b) moderate HL: 41–60 decibels, and (c) severe HL: 61–80 decibels. Profound HL or deafness: more than 81 decibels. A hearing impairment is typically defined as a loss of more than 40 decibels.^19^

The second part of the assessment focused on depression, anxiety, and social isolation using the Hospital Anxiety and Depression Scale (HADS) questionnaire. HADS consists of 14 questions, seven each for anxiety (HAD-A) and depression (HADS-D). Respondents answer on a 4-point Likert scale, and each subscale has scores ranging from 0 to 21. The total scores can range from 0 to 42, with higher scores indicating more severe symptoms. The scores are categorized as follows: normal (0–7), mild distress (8–12), moderate distress (11–14), and severe distress (15–21). It’s important to note that higher scores suggest more severe symptoms.^20^ The Persian version of the HADS was utilized, validated with Cronbach’s alpha coefficients of 0.86 for the HADS-D subscale and 0.78 for the HADS-A subscale.^21^ The specificity and sensitivity for the HADS-A were 0.78 and 0.9, respectively, and for the HADS-D, they were 0.9 and 0.79, respectively.^22^ We also used the Persian version of the 18-item Lubben Social Networks Scale (LSNS-18) to assess social isolation.^23^ The scale underwent evaluation for content and face validity using qualitative methods. It demonstrated strong reliability, with Cronbach’s alpha at 0.82 and an interclass correlation coefficient (ICC) of 0.85.^23^ The scale also demonstrated better fit with a three-factor model compared to a single-factor model, confirming its construct validity. The Persian LSNS-18 is a valid and reliable tool for evaluating social networks within the Persian-speaking community.^23^ The scales were adapted into Dari, which is a mutually intelligible variety of Persian.^17^ Language experts confirmed the equivalence of concepts in the questionnaire. Cultural validation was conducted to ensure appropriate wording and minimize potential misinterpretations due to cultural differences in thinking. Trained doctors conducted the interviews and provided explanations for clarification as needed regarding the concepts in the questions. The LSNS-18 subscale evaluated the size and supportiveness of the network, with scores ranging from 0 to 30. The size of the social network was categorized as follows: severely isolated (3 or less), isolated (>3, =<6), low social ties (>6, =<9), socially connected (>9, =<12), and strong social network (>12). It is well validated in aging samples.^24^

### 2.2. Statistical analysis

Descriptive statistics, such as frequency, percentages, mean, and standard deviation (SD), were used to provide a summary of the sociodemographic and baseline characteristics of the participants. Continuous and categorical variables were compared using the Chi-square test and the *t*-test, respectively. Socioeconomic status variables, including age, sex, educational level, marital status, and occupation, were considered as confounding variables in our study. These factors were adjusted for in the analysis of social isolation, anxiety, and depression because they were identified as potential influences that could affect the study results. Univariate linear regression analyses were performed to identify any associations between social isolation, anxiety, depression, and HL. Variables that showed a significant association with the outcome of interest (with a *p*-value of less than 0.05) in this analysis were included in logistic regression analysis models. Separate models were developed for each study outcome to examine the influence of demographic factors. The logistic regression analysis calculated odds ratios and their corresponding 95% confidence intervals (CIs). Statistical analysis was carried out using the SPSS 20.0 software package (SPSS, Chicago, IL).

## 3. Results

A total of 226 participants were enrolled in the study, comprising 107 (47.3%) males and 119 (52.7%) females. The majority of participants were married (n = 194, 85.8%) and illiterate (n = 142, 62.8%). Additionally, 38.9% (n = 88) were unemployed, 37.2% (n = 83.8) were smokers, 40.2% (n = 94) experienced anxiety, 32.7% (n = 74) reported social isolation, and 25.2% (n = 57) experienced depression. In terms of clinical characteristics, 15.5% (n = 35) had diabetes, and 16.4% (n = 37) had hypertension. A detailed presentation of sociodemographic and clinical characteristics for all participants is available in Table 1.

Table 2 presents information about the association between sociodemographic and clinical characteristics and HL among participants. The mean age of participants with HL was 65.5 ± 12.3 years, compared to 62.6 ± 10.8 years for those without HL. There are more female participants with HL (58.7% vs. 41.3%) than male individuals with HL. Compared to participants without HL, a higher percentage of participants with HL (93.5% vs. 83.9%) is married. The percentage of participants with HL who are unemployed is higher than that of participants without HL (54.3% vs. 35.0%). HL was found to have a statistically significant association with smoking (p = 0.001), and HL showed significant associations with anxiety and depression (p = 0.008 and p = 0.011, respectively). A significant association was also observed between HL and social isolation (p = 0.016). Diabetes mellitus was also associated with HL (p = 0.006), and a significant association existed between HL and hypertension (p = 0.008).

The results of the logistic regression analyses are presented in Table 3. Participants with HL were more likely to have depression (OR = 2.16; 95% CI: 1.05–4.44), social isolation (OR = 2.87; 95% CI: 1.44–5.70), and anxiety (OR = 2.62; 95% CI: 1.21–5.67) compared to participants in the no hearing loss (No-HL) group.

## 4. Discussion

This study investigated the association between HL, depression, anxiety, and social isolation among adult outpatients aged 50 years and older in Afghanistan. The findings of this study supported the hypothesis that HL would be linked to poor social isolation and mental health outcomes.

Our findings are consistent with other studies that have shown higher HL for males than females,^25^ which increased with age.^26^ The present study found that participants with HL were more likely to be smokers, have diabetes, or have hypertension compared to those in the No-HL group. These results are comparable to those found in previous studies.^27,28^ These associations were found to be statistically significant, indicating that smoking, hypertension, and diabetes were identified as contributing factors to HL.^28^

The present study found that HL was associated with depression. These results are comparable to those found in a previous study among patients in the Guangzhou Health and Happiness Association for the Respectable Elders, China.^29^ The correlation between HL and depression that has been reported may be explained by various factors. Psychological distress and depression may be exacerbated by HL’s effects on social engagement and loneliness, as well as its effects on modifications to brain structure and function.^30^ Sensory deprivation has also been suggested as a possible cause of depression.^30^ Health care professionals need to be more aware of the link between HL and poor mental health, given the correlations we have found between HL mental health symptoms and poor social isolation.

Findings from the present study indicated that HL is independently associated with greater odds of anxiety symptoms. The data agree with 1,797 patients at the Tinnitus Clinic in Soree Ear Hospital in South Korea.^31^ Results from previous studies have suggested some possible mechanisms linking HL with anxiety. It is likely that microvascular disease has been linked to HL loss and poor mental health.^32^ There is also a suggestion that anxiety may be influenced by sensory deprivation.^32^

In the present study, we observed that individuals with HL experienced significantly greater social isolation compared to those without HL. The results described in the present study are consistent with an earlier investigation of middle-aged and older adults among participants in primary health care in Brazil.^33^ It is likely that, due to the stigma associated with HL and their fear of communication difficulties, they may also lack the confidence to approach new people and build new relationships.^34^ People with HL had much smaller social networks, and such behavior could be attributed to their small personal networks.^35^ The underlying reasons for these results could be that HL has a strong impact on a person’s wellbeing because it is linked to social withdrawal and social and emotional loneliness.^6^

The findings from this study have significant implications for clinical practice, health policy, and future research. Clinically, the results underscore the importance of a personalized approach when prescribing hearing aids, taking into account each individual’s sociodemographic background and mental health needs. Our study’s findings contribute to a better understanding among health professionals and clinicians of the association between depression, anxiety, social isolation, and hearing status. Recognizing these broader effects highlights the importance of treating HL not only for auditory improvement but also for enhancing mental health and social well-being. Research suggests that using hearing aids holds promise in reducing loneliness, mitigating social isolation, and alleviating depression symptoms in older adults with varying degrees of HL.^36^ Zhang et al. (2024) found that using hearing aids was associated with lower rates of depression and reduced mental health needs, suggesting that hearing aids could potentially prevent mood disorders and decrease the need for primary mental healthcare services.^37^ Furthermore, to improve the impact of our findings, we propose future research directions. These include exploring the longitudinal effects of hearing aid use on mental health outcomes and evaluating the cost-effectiveness of HL interventions in healthcare settings. Such efforts aim to highlight the practical implications of our findings for clinical practice and policy.

West (2017) showed that HL is a persistent stressor in people’s lives. The amount of social support they receive influences their response to this stress, affecting their mental health.^38^ HL can lead individuals to become less socially active and feel less supported by their social circles, potentially leading to social withdrawal and emotional loneliness.^38^ While our study has identified significant associations between HL and conditions like depression, anxiety, and social isolation, further investigation into the underlying mechanisms is warranted. Potential mediators, such as social support or cognitive function, and moderators, including age or coping strategies, could elucidate how HL influences mental health outcomes. For instance, understanding how social isolation mediates the relationship between HL and depression could inform interventions aimed at improving social interactions for individuals with hearing impairments. Similarly, exploring moderators like age might reveal differential impacts of HL on mental health across different life stages. By exploring these mechanisms, future research can provide deeper insights into the complex interplay between HL and mental health, ultimately guiding more targeted interventions and improving overall well-being for affected individuals.

Our study examined how HL affects mental health outcomes, drawing parallels with the “depreobesity” phenomenon. Like the association between obesity and depression, influenced by environment and genetics,^39^ we explored how chronic stressors such as HL interact with depression, anxiety, and social isolation in middle-aged and older adults. Factors like socioeconomic status and genetics also shape these links, similar to patterns seen in “depreobesity."^39^ By highlighting these associations, our study deepens understanding of how chronic conditions, like sensory impairments, impact mental health. These insights are crucial for developing preventive strategies and guiding clinical care in various healthcare settings.

## 5. Limitations and Strengths

The main strength of our study is that it adds to the current body of literature by highlighting the potential link between HL and psychological outcomes in an outpatient clinic that serves as a referral research center in Afghanistan.^18^ However, our study has some limitations. First, the sample size was based on data from a single center. Second, the sample was not randomly selected, thereby restricting overall statistical power. Additionally, we did not measure the magnitude of associations or effect sizes, which could offer valuable insights into the clinical significance of our findings. This will be acknowledged as a limitation in our study. We will include this as a limitation of the study. Finally, the cross-sectional study design means it is difficult to make causal inferences about the data.

## 6. Conclusion

This study’s findings suggest that there is an independent association between HL, depression, anxiety, and social isolation. Hearing aids can help alleviate some of these effects. This study’s findings suggest that there is an independent association between HL, depression, anxiety, and social isolation. Hearing aids can help alleviate some of these effects. Future research, particularly longitudinal studies, is crucial to further elucidate the causal relationships between HL and mental health outcomes. These findings emphasize the importance of screening all adults of middle age and older for hearing impairments, as well as corrective interventions and support, aiding in the translation of emerging knowledge into policy and practice.

## Acknowledgments

This project was subsidized by the Terumo Life Science Foundation.

## Conflict of Interest Statement

None.

## Figures and Tables

**Table 1.** The frequency distribution of sociodemographic and clinical characteristics among study participants.

**Characteristics**	**Total *n* (%)**
Age mean (SD)	63.2 (11.2)
Gender	
Male	107 (47.3)
Female	119 (52.7)
Marital Status	
Married	194 (85.8)
Single	24 (10.6)
Others	8 (3.5)
Level of education	
Illiterate	142 (62.8)
Primary/private education	16 (7.1)
Secondary	37 (16.4)
High school or more	31 (13.7)
Occupation	
Employed	57 (25.2)
Unemployed	88 (38.9)
Housewife	49 (21.7)
Other types of jobs	32 (14.2)
Smoking status, yes	84 (37.2)
Anxiety, yes	94 (40.2)
Depression, yes	57 (25.2)
Diabetes mellitus, yes	35 (15.5)
Social isolation, yes	74 (32.7)
Hypertension, yes	37 (16.4)

SD: standard deviation.

**Table 2.** Sociodemographic and clinical characteristics of study participants by their hearing status.

**Variables**	**HL (*n* = 46)**	**No-HL (*n* = 180)**	***p*-Value**
Age (in years), mean (SD)	65.5 (12.3)	62.6 (10.8)	0.055
Gender (males/females, %)	41.3/58.7	48.9/51.1	0.41
Marital status, no (%)			0.256
Single	2 (4.3)	22 (12.2)	
Married	43 (93.5)	151 (83.9)	
Others	1 (2.2)	7 (3.9)	
Level of education, *n* (%)			0.861
Illiterate	29 (63.0)	113 (62.8)	
Primary/private	3 (6.5)	13 (7.2)	
Secondary	9 (19.6)	28 (15.6)	
High school or higher	5 (10.9)	26 (14.4)	
Occupation, no (%)			0.017
Employed	7 (15.2)	50 (27.8)	
Unemployed	25 (54.3)	63 (35.0)	
Housewife	5 (10.9)	44 (24.4)	
Other types of jobs	9 (19.6)	23 (12.8)	
Smoking status no (%) current	29(63.0)	55(30.6)	0.001
Anxiety (%), yes	15 (32.6)	31 ( 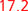 )	0.013
Social isolation no (%), yes	24 (52.2)	50 (27.8)	0.002
Depression no (%), yes	18(39.1)	39 (21.7)	0.014
Diabetes mellitus (%), yes	20(43.5)	15 (8.3)	0.001
Hypertension (%), yes	25 (54.3)	12 (6.7)	0.001

HL: hearing loss, No-HL: no hearing loss group, SD: standard deviation.

**Table 3.** Association between hearing loss, depression, anxiety, and social isolation.

**Variables**	**%**	**OR**	**95% Cl**	***p*-Value**
Anxiety				
No	67.4	Ref		
Yes	32.6	2.62	1.21–5.67	0.014
Depression				
No	60.9	Ref		
Yes	39.1	2.16	1.05–4.44	0.036
Social Isolation				
No	47.8	Ref		
Yes	52.2	2.87	1.44–5.70	0.003

Ref: reference, OR: odds ratio, CI: confidence interval, %: percentage.
